# How Will Climate Warming Affect Non-Native Pumpkinseed *Lepomis gibbosus* Populations in the U.K.?

**DOI:** 10.1371/journal.pone.0135482

**Published:** 2015-08-24

**Authors:** Grzegorz Zięba, Michael G. Fox, Gordon H. Copp

**Affiliations:** 1 Salmon and Freshwater Team, Centre for Environment, Fisheries and Aquaculture Science, Lowestoft, Suffolk, United Kingdom; 2 Environmental and Resource Studies Program and Department of Biology, Trent University, Peterborough, Canada; 3 Centre for Conservation Ecology and Environmental Science, Bournemouth University, Poole, Dorset, United Kingdom; Estación Biológica de Doñana, CSIC, SPAIN

## Abstract

Of the non-native fishes introduced to the U.K., the pumpkinseed is one of six species predicted to benefit from the forecasted climate warming conditions. To demonstrate the potential response of adults and their progeny to a water temperature increase, investigations of parental pumpkinseed acclimatization, reproduction and YOY over-wintering were carried out in outdoor experimental ponds under ambient and elevated water temperature regimes. No temperature effects were observed on either adult survivorship and growth, and none of the assessed reproductive activity variables (total spawning time, spawning season length, number of spawning bouts) appeared to be responsible for the large differences observed in progeny number and biomass. However, it was demonstrated in a previous study [Zięba G. et al., 2010] that adults in the heated ponds began spawning earlier than those of the ambient ponds. Ambient ponds produced 2.8× more progeny than the heated ponds, but these progeny were significantly smaller, probably due to their late hatching date, and subsequently suffered very high mortality over the first winter. Pumpkinseed in the U.K. will clearly benefit from climate warming through earlier seasonal reproduction, resulting in larger progeny going into winter, and as a result, higher over-winter survivorship would be expected relative to that which occurs under the present climatic regime.

## Introduction

A crucial question posed by scientists, environmental managers and government officials focuses on how non-native species will respond to the predicted increases in temperature associated with climate warming, e.g [1, 2, 3]. For inland waters of England, the increase is expected to be 2–3°C and to be accompanied by more intensive and more frequent floods and spates [4]. Of particular concern are the potential effects of climate warming on the establishment and recruitment of freshwater fishes [5], which are capable of causing major shifts in ecosystem function (e.g. common carp *Cyprinus carpio*; [6]) or can disrupt native species through parasitism and reproductive interference (e.g. topmouth gudgeon *Pseudorasbora parva*; [7, 8]. The ability of an introduced species to establish populations and expand in novel environments is largely dependent on its ability to produce young that can survive and successfully reproduce over successive generations under these new conditions.

As with many ectotherms, fish have a preferred temperature range that affects bioenergetic functions such as food consumption rates and somatic growth [9, 10], as well as life history traits, e.g. age at maturity, size at maturity and generation time [11]. Temperature can also affect the seasonality of reproduction, and in multiple batch spawners it can also affect the length of its reproductive season [12]. In middle-to-high latitude fishes, an early onset of reproduction can result in the production of larger eggs, potentially leading to a longer period of first year growth and consequent larger body size prior to the first winter, e.g. [13, 14]. Similarly, in non-native populations of pumpkinseed *Lepomis gibbosus* (L.), a North American centrarchid, the growth rate of immature age-1 females in their second growing season has been found to be positively related to thermal degree-days in the spring, with elevated reproductive effort of age-1 females in warmer water bodies [15]. Given that size-dependent processes such as size-selective predation and rate of over-winter energy loss can potentially affect the survival rate of the young of many fish species, e.g. [16, 17, 18, 19], a change in temperature regime such as would occur from climate warming could affect the relative survival and recruitment rates of native and non-native fishes adapted to different temperature regimes.

Of the existing non-native fish species in England and Wales, six have been identified as likely to benefit from climate warming [5]. This includes the pumpkinseed, which is currently considered to be non-invasive in the U.K. [4, 20] despite being established in most European countries and being invasive mainly in southern latitudes [21, 22]. Colonisation success of non-native animals such as the pumpkinseed is determined, among other factors, by dispersal rate, which strongly depends on offspring number and their fitness [23]. In the case of non-native pumpkinseed in Southern England, the number of potential colonisers from existing sites is likely to increase with increasing discharge, e.g. during spates and floods [4]. Although different life stages have different characteristics as propagules, it is mainly pumpkinseed larvae and young-of-year (YOY) juveniles that are likely to dominate in river drift, e.g. [24] and outflows from connected water bodies [4].

According to Sargent *et al*. [25], organisms should spend their life history in a region where the somatic growth rate is higher than the mortality rate, therefore each dispersal attempt towards an unknown environment seems hazardous. When this happens, however, the number of offspring and especially their fitness may be crucial for survival over the first winter. Being susceptible to high mortality rates [26], juvenile fishes have received particular attention, and several concepts have attempted to predict the optimal size for offspring [27] or the dependency of mortality on somatic growth [28]. Offspring size generally depends on growth conditions and may even increase due to higher investment when environmental conditions worsen [29], or take the form of a sigmoid relationship due to intraspecific competition [30]. Mortality varies in magnitude and usually decreases with increasing body size [31], or where parental guarding of eggs and larvae is present. However, food availability and temperature play a crucial role in shaping the development of offspring size through somatic growth or mortality rates [23].

Native pumpkinseed populations are known to occupy, and reproduce in, both lotic and lentic ecosystems, but reproduction of non-native populations in Europe is less common in rivers, and in England reproduction is currently limited to still waters [21]. Nonetheless, non-native pumpkinseed populations in Europe exhibit considerable plasticity in habitat use, diet [32] and life-history traits [21, 33], though recent research on repatriated pumpkinseed suggests that European populations are less phenotypically plastic than native populations [34, 35, 36]. Although the number of studies on the pumpkinseed’s response to climate warming is increasing [37, 4, 15, 38], additional work is needed to understand the mechanisms by which pumpkinseed could become more invasive [21], such as potential shifts in the timing and scale of life-history events [39] and climate niche shifts [40]. This is of particular relevance to shallow inland waters, such as ponds, which are now known to support disproportionately high aquatic biodiversity [41] and are believed to be particularly susceptible to the future warmer conditions [39].

The aim of the present study was to examine the effect of thermal warming associated with climate change projections on the growth and survivorship of YOY pumpkinseed in southern England. Using outdoor experimental ponds in which water temperature was manipulated to simulate future (warmer) climatic conditions, the specific objectives of the study were to compare ambient and heated temperature regimes in terms of: (i) growth of adult pumpkinseed during the reproductive period; and (ii) number and body size of YOY pumpkinseed produced under the two thermal regimes prior to winter. We also discuss implications of our results for understanding the process of pumpkinseed dispersal under the climate warming scenario predicted for the U.K.

## Materials and Methods

The investigation was conducted with permission of the land owners in six independent, outdoor experimental ponds situated on private land at Tanyard Fishery (Furners Green, Uckfield, East Sussex, TN22 3RL, England; Latitude 51:01:07°N, Longitude 0:00:47E). The ponds are of 5 × 5 m surface area [37, 38] and have variable bathymetry: a 1 m wide shallow shelf along one side for male pumpkinseed to nest (mean depth 0.5 m) and a deeper (1.2 m) refuge/resting area for females. When constructed, each pond was lined with a sheet of rubberised plastic and was fitted with an identical self-contained water recirculation system (P2500, Bladgon, U.K.). Water was pumped into a fibreglass cistern (0.2 m^3^) at a maximum rate of 2400 L h^-1^, with water overflowing via a pipe back into the pond. In the three heated ponds, the cisterns were fitted with floating, electric styrofoam heaters (Velda, NL, 750 W) to maintain water temperatures at 2–3°C above that of the three (ambient temperature) ponds. Pond temperatures were monitored continuously using TinyTag “Aquatic 2” temperature loggers (Gemini Data Loggers Ltd, U.K.). Gravel-filtered water was taken from an adjacent holding pond to replace any natural loss of water due to evaporation. The study was undertaken in two phases: (I) thermal acclimation of adults; (II) reproduction (production of offspring).

In Phase I, 120 pumpkinseed of indiscernible gender were captured by electrofishing on 7 November 2008 from one of the nearby commercial angling ponds. The fish were anaesthetised by immersion in 2-phenoxy ethanol (1 mL L^-1^ of anaesthetic bath) and individually-tagged as per Stakėnas *et al*. [42] with passive integrated transponder (PIT) tags (Wyre Micro Design Ltd, U.K.: 2 × 12 mm, 0.1 g each), which were inserted manually using a sterilized PIT tag needle to penetrate the body cavity in the ventral area between the pelvic fins and anus. These tags enabled us to determine changes in total body length (*L*_T_) and weight when the adults were collected at the end of this phase of the experiment. To reduce the risk of infection, a mixture of antibiotic (Cicatrine) and adhesive (Orahesive) powders was applied to the insertion point, and the fish were placed in clean, aerated water to recover.

Immediately after their full recovery from anaesthesia, 20 pumpkinseed of indiscernible gender were released into each pond. Fish were inspected visually each day and fed twice per week *ad libitum* (until the fish stopped active feeding) with live white maggots, and every other day with frozen chironomid larvae (16–24 g per pond, depending on the season). Feeding was curtailed in all ponds on 14 December 2008, when the mean daily water temperature dropped below 5°C in the ambient ponds. Although there was some formation of ice during the winter period, water circulation from the pumps kept the ponds from freezing over entirely, even on the coldest days. Feeding recommenced on 20 March 2009, when the temperature reached 5°C in the warmest pond.

Individual adults were removed from the ponds on 14 May 2009, measured for *L*_T_ and wet weight (Wt), and their gender was determined by visual estimation of secondary sexual characteristics and confirmed after the experiment by dissection. Mean *L*_T_ of pumpkinseed did not differ significantly among treatments (ANOVA, n = 120, *P* = 0.10). In Phase II of the study, 42 males (*L*_T_ 101–128 mm) and 42 females (*L*_T_ 104–129 mm) were selected from the 120 over-wintered adults, with 14 randomly-selected pumpkinseed (seven males, seven females) stocked back into a pond of the same temperature treatment. A number of pumpkinseed spawning metrics were monitored during Phase II and those results have been previously reported in Zięba *et al*. [37].

The experimental ponds were drained on 14 October 2009, and all recovered YOY were held separately in aerated containers (one per pond). All recovered individuals except for a sample used in a subsequent over-winter survival study (see SI Appendix 1) were euthanized according to a Home Office licensed regulated procedure (overdose of 2-phenoxy-ethanol), chilled to frozen, then partially defrosted and preserved in 4% formalin. These individuals were later counted, measured (*L*_T_, nearest mm) and weighed (nearest 0.01 g). The investigations were carried out under licence from the UK Home Office, accompanied by the necessary licences, consents and/or derogations as regards the Import of Live Fish (England and Wales) Act 1980 and related legislation.

## Data Analysis

To assess differences in biomass between thermal regimes for adult pumpkinseed at the start of the study, and then somatic growth during phases I (acclimation) and II (reproduction), the data for the 42 males and females used in Phase II (the reproductive study) were compared using Analysis of Variance (ANOVA), with treatment as the fixed effect and pond replicate as the random effect. One-way ANOVA was used to test for differences between the two thermal regimes in terms of YOY number and total biomass in each pond at the end of Phase II (October 2009) as well as differences between thermal regimes in the mean *L*_T_ and mass of individual YOY, using means by pond from the sub-sample of recovered individuals. To determine whether or not the amount of reproductive activity in a pond could explain among-pond variation in the number or total mass of YOY recovered, Pearson correlations were conducted using three indicators of reproductive activity generated for each sex (total spawning time, length of spawning season, number of spawning bouts) from the adult telemetry data previously described in Zięba *et al*. [37]. These variables were examined individually in Analysis of Covariance, using temperature treatment (ambient, heated) as a main effect and the reproductive variable as the covariate. Date of first spawning was not used in this analysis because it had already been considered in a previous study [37]. he number, total mass, and mean *L*_T_ and weight of YOY recovered were log_e_-transformed prior to all of these analyses to correct for heteroscedasticity in the raw data.

We tested for differences between treatments in YOY body condition index to infer changes that may influence over-winter survival. For this, we used Analysis of Covariance on log_e_-transformed *L*_T_ and weight data on YOY recovered prior to winter (October 2009), with weight of the fish as the dependent variable, *L*_T_ as the covariate and the interaction between *L*_T_ and treatment as variables in the model. Pond within treatment was included as a random effect in the mixed model to account for the treatment replicates, and effects were estimated in the model with the Restricted Maximum Likelihood Method using the JMP 11 statistical program. While this method does not fully account for changes in the length-weight relationship as an animal grows [43]; it was used to detect treatment differences rather than temporal changes in body condition associated with growth.

## Results

**Fig 1:**
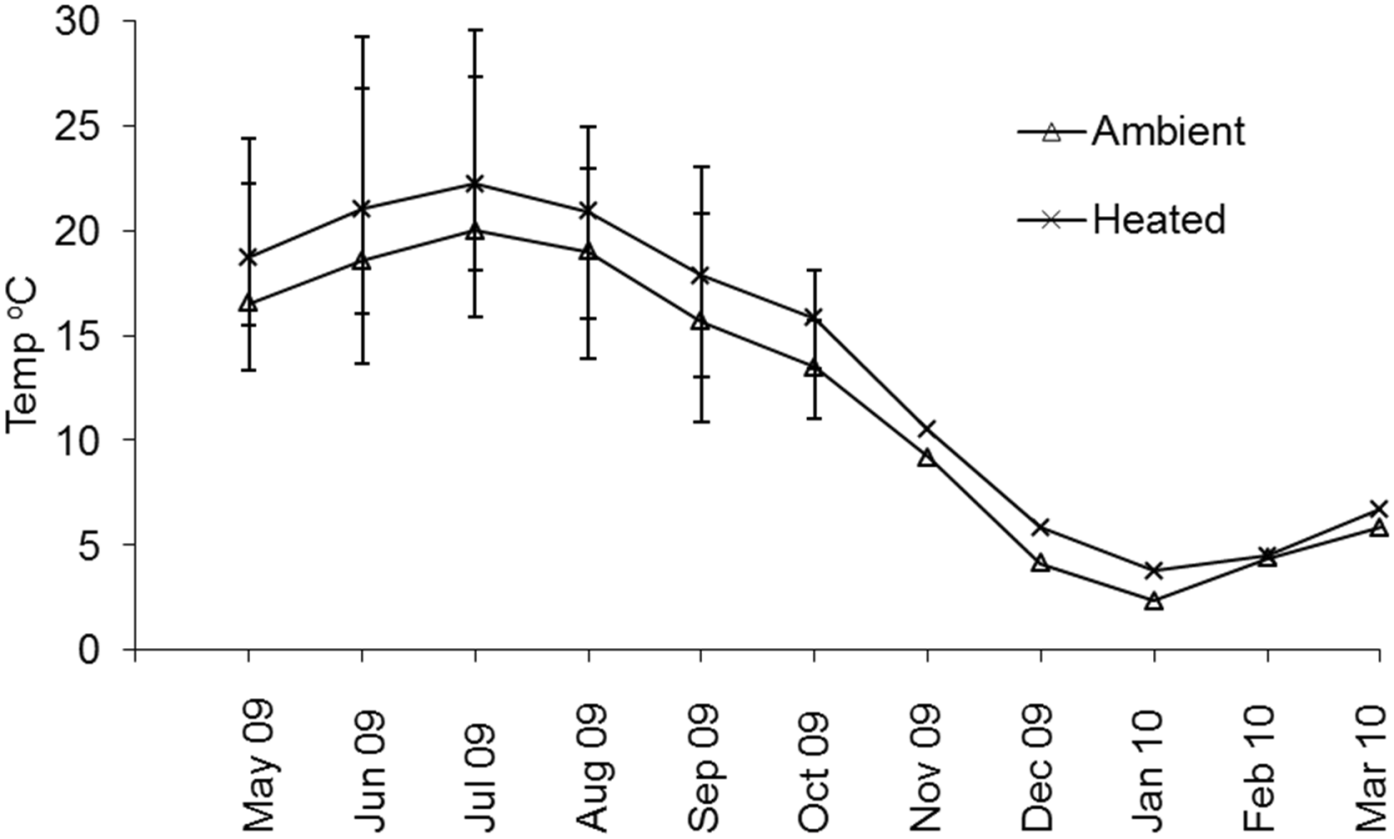
Mean and standard deviation of monthly water temperatures in the three ambient and heated experimental ponds (England) during the study period in 2009–2010.

The mean monthly water temperatures in the three ambient and three heated ponds differed over the entire investigation: min–max: 1.0–27.4°C, 1.8–30.7°C, respectively (Fig 1). The mean difference was intended to be ≈ 2–3°C, and this was maintained throughout the study except in mid-February 2010, when a short-term (six days) heating-system failure occurred. The adult pumpkinseed acclimated and used in reproduction under ambient and elevated temperatures were similar in *L*_T_ and weight at the outset of the study (Table 1). Males and females in both treatments grew in size and gained mass during Phase I (acclimation) and Phase II (reproduction), with most of the growth occurring in Phase II, when temperatures were warmer and artificial feeding occurred throughout. There were no significant differences in *L*_T_ or weight of the adults between treatments in either sex at the start of the acclimation period, or at the start and end of Phase II (*F*_1,4_ < 3.53; *P* > 0.13 in all comparisons).

**Table 1:** Mean total length (*L*_T_) and mass of PIT-tagged adult male (*n* = 42) and female (*n* = 42) pumpkinseed used in the reproductive portion of the study, from the initiation of the acclimation period (Nov. 2008) to the end of the reproductive period (Oct. 2009). Each treatment consisted of *n* = 7 males and *n* = 7 females.

	*L*_T_ (mm)			Mass (g)		
	Nov 2008	May 2009	Oct 2009	Nov 2008	May 2009	Oct 2009
(A) Males						
Ambient 1	110.1	110.7	123.4	24.1	28.1	43.0
Ambient 2	113.1	113.9	126.4	26.9	30.7	45.8
Ambient 3	116.1	116.7	132.3	28.0	33.2	52.0
Mean:	113.1	113.8	127.4	26.3	30.7	46.9
Heated 1	114.0	114.9	128.0	27.1	32.0	44.1
Heated 2	113.6	114.0	128.0	25.3	29.8	42.9
Heated 3	113.1	115.4	128.3	25.8	34.5	47.5
Mean:	113.6	114.8	128.1	26.1	32.1	44.8
(B) Females						
Ambient 1	112.6	114.0	125.9	25.3	29.6	44.4
Ambient 2	106.4	110.0	121.0	22.5	25.9	39.9
Ambient 3	111.4	112.1	123.6	24.6	28.7	42.9
Mean	110.1	112.0	123.5	24.1	28.1	42.5
Heated 1	114.1	114.6	129.4	26.0	30.0	45.3
Heated 2	110.9	112.3	124.4	24.2	29.7	40.6
Heated 3	113.7	115.6	128.1	26.6	35.1	47.6
Mean:	112.9	114.1	127.3	25.6	31.6	44.5

At the end of Phase II (reproduction), approximately 2.8× more YOY were recovered in the ambient ponds than in the heated ponds, but total biomass of YOY was greater in the heated ponds (Table 2). Neither difference was statistically significant (number: *F*_1,4_ = 1.87; *P* = 0.24; mass: *F*_1,4_ = 1.68; *P* = 0.27), but the YOY recovered from the heated ponds were significantly larger than those from the ambient temperature ponds (*L*_T_: *F*_1,4_ = 30.3; *P* = 0.0053; mass: *F*_1,4_ = 33.2; *P* = 0.0045). None of the reproductive activity variables (total spawning time, spawning season length, number of spawning bouts) could explain a significant portion of the variability in number or mass of YOY recovered at the end of the reproductive phase, either alone or in combination with the temperature treatment. The strongest of these relationships was between mean spawning time and number of YOY recovered, but even this relationship was not significant (*P* = 0.16 for each sex). An analysis of body condition indices of YOY prior to winter indicates that while pumpkinseed born in heated ponds were slightly thinner relative to their length than those born in the ambient temperature ponds, the difference between temperature treatments was not significant (Table 3). The significant length × treatment interaction was due to the body condition index in YOY in the heated ponds being lower than that of the ambient ponds in the smallest individuals, but higher in the larger individuals.

**Table 2:** Numbers (*n*) of YOY pumpkinseed recovered in experimental ponds (East Sussex, England) and their overall weights (Wt) in each pond, with mean, maximum (Max.) and minimum (Min.) for total length (*L*_T_) and body weight by temperature regime prior to and after the winter of 2009–2010.

Time period			Wt in	*L*_T_ (mm)			Weight (g)		
Temp.	Pond	*n*	Pond (g)	Mean	Min.	Max.	Mean	Min.	Max.
End of Phase II (October 2009)									
Ambient	1	665	78.53	19	10	38	0.12	0s.01	0.93
Ambient	2	1567	135.06	17	8	39	0.09	0.01	1.05
Ambient	3	228	34.13	21	11	36	0.15	0.03	0.62
Totals and means:		2460	247.72	**19** ^**¥**^			**0.10** ^**†**^		
Heated	1	312	99.68	27	16	51	0.32	0.07	2.12
Heated	2	255	112.51	31	23	55	0.44	0.16	2.64
Heated	3	299	174.75	33	24	63	0.58	0.19	4.45
Totals and means:		866	386.94	**30** ^**¥**^			**0.45** ^**†**^		

Mean values in bold and annotated with the same symbol (^**¥**^ or ^**†**^) are significantly different between temperature treatments (Students’ *t*-test, *P* > 0.0001).

**Table 3:** Results of Analysis of Covariance, testing for differences in body condition index (wet weight relative to total length) between young-of-the-year pumpkinseed recovered from ambient temperature and heated ponds in October 2009. The analysis included a pond-within-treatment random effect to account for treatment replicates (not shown).

Fixed effects	df	*F*	*P*
Total length	1, 2 991	42.01	< 0.001
temperature treatment	1, 5.06	3.97	0.102
length × treatment	1, 2 991	28.9	< 0.001

## Discussion

The success of non-native fishes, e.g. in terms of the number of newly-established populations in novel environments, may be the consequence of the species’ adaptability and the potential for dispersal of both juveniles and adults [38, 44, 45]. Our results suggest that pumpkinseed populations subjected to elevated water temperatures, which may result from either a long-term trend associated with climate warming [1, 2, 3] or an unexpected short-term climatic event (e.g. a heat wave; [46]), will not necessarily have a direct effect on adult somatic growth (Table 1) and/or fecundity. However, a longer-term temperature increase, such as under the predicted climate change conditions, may result in a population increase in this non-native species as a consequence of faster somatic growth of progeny and consequently, higher over-winter survival. None of the spawning effort indices, which is described in Zięba *et al*. [37], differed significantly under ambient and heated conditions, or were responsible for differences in YOY number and mass at the end of the study. However, female pumpkinseed in the heated ponds began their reproductive season significantly (8 days) earlier than those in the ambient ponds [37]–a phenomenon observed in lakes situated along a latitudinal gradient [47]. Early spawning provides a compensatory mechanism in the face of unpredictable conditions, assuring that at least some progeny will experience the optimal conditions for growth [14]. In fact, earlier spawning provided the pumpkinseed progeny in the heated pond a longer, warmer first-year growing season. This translated into greater pre-winter size (in both *L*_T_ and weight) than that of progeny from the ambient ponds (Table 2; Fig A in S1 File).

Elevated water temperature increases pumpkinseed invasion success in warmer parts of non-native occurrence range (e.g. southern Europe) through faster juvenile growth and earlier maturity [21, 48]. Lower temperatures may have the opposite effect. Temperature profiles during the spawning period suggest that in some northern parts of the native range [49] pumpkinseed offspring may experience higher temperatures than in the U.K. Spring and summer temperatures are relatively low in the U.K., which may be the underlying reason why the species is currently not invasive in this part of the species’ introduced range [4, 20, 21].

The earlier-hatched pumpkinseed progeny in the heated ponds had a longer period during which fish could accumulate mass before winter than could the later-hatched progeny in the ambient ponds, resulting in larger body size. Over-wintering of these YOY until the following spring in an ambient and a heated pond demonstrated that the larger-bodied individuals produced in the heated ponds experienced a much higher survival rate (S1 File), and that the smallest individuals in the ambient pond showed the highest mortality rate (Table A and Fig A in S1 File). Although the over-winter survival results are not definitive because of the lack of replication, they are consistent with several previous studies of centrarchids, which have showed that YOY hatched earlier in the spawning season make a disproportionately high contribution to the entire year-class [14, 50, 51, 52]. An over-winter survival advantage for larger-bodied (early born) progeny has also been reported in a European cyprinid, the roach *Rutilus rutilus* [53], as well as several North American species [16], including fathead minnow *Pimephales promelas* [54], pumpkinseed [55], and bluegill *Lepomis macrochirus* [14].

Higher overall water temperature is known to stimulate growth in pumpkinseed, e.g. [56], which in our experiment was evident in the higher mean and total biomass of juveniles in the heated ponds. Although the adult pumpkinseed were fed *ad libitum* at all treatments, we cannot exclude the possibility that higher energy demand in heated sites caused by increased metabolic demand resulted in food limitation and subsequent cannibalism on YOY. Copp *et al*. [57] found that for pumpkinseed inhabiting an artificial pond in Cottesmore (south-eastern England, < 50 km from the present study), limited food resources resulted in conspecific cannibalism and intensive egg predation. The observed distinct size distribution pattern within populations inhabiting the warmer experimental ponds, and reduced number of YOY (particularly of the smallest size classes) might have thus been the result of not only higher growth rate, but also intraspecific competition for food, leading to predation. As noted in previous studies with Eurasian perch *Perca fluviatilis*, the potential effects of adult cannibalism on YOY can be complex, and they may include the restriction of YOY to refuges where feeding and growth are reduced as well as an increase in early-life mortality [58, 59].

In the northern part of their native range, few pumpkinseed < 35 mm *L*_T_ are able to survive their first winter [48, 60]. For coastal embayments of Lake Ontario, the threshold body size for over-winter survival was estimated at 26 mm *L*_T_ [56], based on the body size of individuals caught in the spring. In the present study, a large proportion of pumpkinseed YOY were <26 mm *L*_T_ by the late fall (particularly in the ambient temperature ponds). However, the U.K. experiences much warmer winters than those of the species’ northern native range, where there are long periods when temperatures are colder than the threshold feeding temperature of 8°C suggested by Keast [61] in his winter study. We would therefore expect to see a higher over-winter survival rate of the small-bodied progeny produced in our experiment in both treatments. Nevertheless, the larger body size in YOY reared under elevated temperature conditions are predicted to produce stronger year classes under a future climate warming scenario, and this prediction was supported by our over-winter results.

Aside from body size considerations, a difference of 2°C in winter temperature would result in a shorter period during which water temperatures are below the feeding threshold in pumpkinseed. Murphy *et al*. [56] suggested that the longer available feeding period could explain differences between warm and cool embayments in the occurrence of pumpkinseed YOY in Lake Ontario. A longer period of temperatures that are sufficiently low to prevent feeding can result in faster depletion of energy reserves in YOY during winter (see [62]) and may also increase their osmoregulatory stress (see [63]). Support for these additional effects is provided by the poor body condition index of small YOY that over-wintered in the ambient ponds relative to their conspecifics reared in warmer water (Table B in S1 File).

As predicted by Britton *et al*. [5], the pumpkinseed should benefit from the warmer conditions forecasted for southern England. The results of our study suggest that the immediate mechanism is reproduction earlier in the season, leading to larger body size and enhanced fitness of YOY at the end of their first summer, leading to greater over-winter survival and subsequent earlier maturity and higher fecundity, e.g. [64, 65]. However, to be more certain of the broad applicability of our results, the study should be replicated in more of the habitat types that the species inhabits in the U.K., with additional temperature treatments and with other source populations.

For the U.K., the climate warming scenario, e.g. [1, 2, 3], predicts not only higher temperature, but also higher risk of floods and therefore higher probability of new pumpkinseed populations being established [4]. This enhanced recruitment will increase the likelihood of higher densities and wider dispersal of pumpkinseed within the U.K., which may, in turn, pose a high risk to native fauna living in waters occupied by this non-native species.

## Supporting Information

S1 File Additional text and supporting figure and tables regarding the study of pumpkinseed recovered after the over-winter period in the experimental ponds. **Fig A in S1 File.** Illustrates the total length-frequency distributions for young-of-the-year pumpkinseed reared in ambient and heated experimental ponds (England) at the end of phases II and III of the experiment. **Table A in S1 File.** Presents the number and total weight of YOY pumpkinseed recovered in an ambient and heated pond following the over-winter period, including mean, minimum and maximum total length and weight values. **Table B in S1 File.** Presents the results of Analysis of Covariance tests for differences in body condition between young-of-the-year pumpkinseed recovered from ambient temperature and heated ponds in March 2010.
(DOCX)

## References

[1] Hulme M, Lu X, Turnpenny J, Mitchell T, Jenkins G, Jones R, *et al*. (2002) Climate change scenarios for the United Kingdom: the UKCIP02 Scientific Report. Tyndall Centre for Climate Change Research, University of East Anglia, Norwich.

[2] IPCC (2007) Summary for policymakers. 1. In: Solomon SQD, Manning M, Chen Z, Manning M, Averyt KB, Tignor M, Miller HL, editors. Climate Change 2007: The Physical Science Basis. Contribution of Working Group I to the Fourth Assessment Report of the Intergovermental Panel on Climate Change. Cambridge University Press, Cambridge and New York, NY. pp. 12–14.

[3] Jenkins GJ, Murphy JM, Sexton DMH, Lowe JA, Jones P, Kilsby CG (2009) UK Climate Projections: Briefing report. Met Office Hadley Centre, Exeter, UK.

[4] Fobert E, Zięba G, Vilizzi L, Godard MJ, Fox MG, Stakènas S, et al (2013) Predicting non-native fish dispersal under conditions of climate change: case study in England of dispersal and establishment of pumpkinseed *Lepomis gibbosus* in a floodplain pond. Ecol Freshw Fish 22: 106–116.

[5] Britton JR, Cucherousset J, Davies GD, Godard MJ, Copp GH (2010) Non-native fishes and climate change: predicting species responses to warming temperatures in a temperate region. Freshw Biol 55: 1130–1141.

[6] Vilizzi L (2012) The common carp, C*yprinus carpio*, in the Mediterranean region: origin, distribution, economic benefits, impacts and management. Fish Manag Ecol 19: 93–110.

[7] Libosvárský C, Baruš CV, Sterba O (1990) Facultative parasitism of *Pseudorasbora parva* (Pisces). Folia Zool Brno 39: 355–360.

[8] Britton JR, Davies GD, Brazier M (2009) Eradication of the invasive *Pseudorasbora parva* results in increased growth and production of native fishes. Ecol Freshw Fish 18: 8–14.

[9] Evans DO (1984) Temperature independence of the annual cycle of standard metabolism in the pumpkinseed. Trans Am Fish Soc 113: 494–512.

[10] Whitledge GW, Hayward RS, Rabeni CF (2002) Effects on temperature on specific daily metabolic demand and growth scope of sub-adult and adult smallmouth bass. J Freshw Ecol 17: 353–361.

[11] Dhillon RS, Fox MG (2004) Growth-independent effects of temperature on age and size at maturity in Japanese medaka (*Oryzias latipes*). Copeia 2004: 37–45.

[12] Jobling M (1995) Reproduction Environmental Fish Physiology. Chapman and Hill, London, UK.

[13] Ware DM (1975) Relation between egg size, growth, and natural mortality of larval fish. Can J Fish Aquat Sci 32: 2503–2512.

[14] Cargnelli LM, Gross MR (1996) The temporal dimension in fish recruitment: birth date, body size, and size-dependent survival in a sunfish (bluegill: *Lepomis macrochirus*). Can J Fish Aquat Sci 53: 360–367.

[15] Masson G, Valente E, Fox MG, Copp GH (2014) Thermal influences on life-history traits and reproductive effort of introduced pumpkinseed sunfish *Lepomis gibbosus* in the River Moselle basin (northeastern France). River Res Appl 31: 563–575.

[16] Toneys ML, Coble DW (1979) Size-related, first winter mortality of freshwater fishes. Trans Am Fish Soc.108: 415–419.

[17] Shuter B J, Maclean JA, Fry FEJ, Regier HA (1980) Stochastic simulation of temperature effects on first-year survival of largemouth bass. Trans Am Fish Soc 109: 1–34.

[18] Post JR, Prankevicius AB (1987) Size-selective mortality in young-of-the-year yellow perch (*Perca flavescens*): evidence from otolith microstructure. Can J Fish Aquat Sci 44: 1840–1847.

[19] Smith RW, Griffith JS (1994) Survival of rainbow trout during their first winter in the Henrys Fork of the Snake River, Idaho. Trans Am Fish Sci 123: 747–756.

[20] Cucherousset J, Copp GH, Fox MG, Sterud E, Van Kleef HH, Verreycken H, et al (2009) Life-history traits and potential invasiveness of introduced pumpkinseed *Lepomis gibbosus* populations in northwestern Europe. Biol Invasions 11: 2171–2180.

[21] Copp GH, Fox MG (2007) Growth and life history traits of introduced pumpkinseed (*Lepomis gibbosus*) in Europe, and the relevance to invasiveness potential In: Gherardi F, editor. Freshwater Bioinvaders: Profiles, Distribution, and Threats. Berlin: Springer pp. 289–306.

[22] Almeida D, Vilizzi L, Copp GH (2014) Interspecific aggressive behaviour of invasive pumpkinseed *Lepomis gibbosus* in Iberian fresh waters. PLoS One 9: e88038 10.1371/journal.pone.0088038 24505367 PMC3914894

[23] Jørgensen C, Auer SK, Reznick DN (2011) A model for optimal offspring size in fish, including live-bearing and parental effects. Am Nat 177: 119–135. 10.1086/657624 21508600

[24] Copp GH, Cellot B (1988) Drift of embryonic and larval fishes, especially *Lepomis gibbosus* (L.), in the Upper Rhône River. J Freshw Ecol 4: 419–424.

[25] Sargent RC, Taylor PD, Gross MR (1987) Parental care and the evolution of egg size in fishes. Am Nat 129: 32–46.

[26] Houde ED (1997) Patterns and trends in larval-stage growth and mortality of teleost fish. J Fish Biol 51 Suppl. A: 52–83.

[27] Smith CC, Fratwell SD (1974) The optimal balance between size and number of offspring. Am Nat 108: 499–506.

[28] Anderson JT (1988) A review of size dependent survival during pre-recruit stages of fishes in relation to recruitment. J Northwest Atl Fish Sci 8: 55–66.

[29] Bashey F (2006) Cross-generational environmental effects and the evolution of offspring size in the Trinidadian guppy *Poecilia reticulata* . Evolution 60: 348–361. 16610325

[30] Brockelman WY (1975) Competition, the fitness of offspring, and optimal clutch size. Am Nat 109: 677–699.

[31] McGurk MD (1986) Natural mortality of marine pelagic fish eggs and larvae: role of spatial patchiness. Mar Ecol Prog Ser 34: 227–242.

[32] Tomeček J, Kováč V, Katina S (2007) The biological flexibility of the pumpkinseed: a successful colonizer throughout Europe In: Gherardi F, editor. Freshwater Bioinvaders: Profiles, Distribution, and Threats. Berlin: Springer pp. 307–336.

[33] Fox MG, Copp GH (2014) Old world vs new world—Life history alterations in a successful invader introduced across Europe. Oecologia 174: 435–446. 10.1007/s00442-013-2776-7 24065557 PMC3897869

[34] Yavno S, Fox MG (2013) Morphological change and phenotypic plasticity in native and non–native pumpkinseed sunfish in response to sustained water velocities. J Evol Biol 26: 2383–2395. 10.1111/jeb.12230 24070018

[35] Yavno S, Fox MG, Vila-Gispert A, Bhagat Y. (2013) Morphological differences between native and non-native pumpkinseed in traits associated with locomotion. Environ Biol Fish 96: 507–518.

[36] Yavno S, Rooke AC, Fox MG (2014) Morphological change and phenotypic plasticity in native and non–native pumpkinseed sunfish in response to competition. Naturwissenschaften 101: 479–492. 10.1007/s00114-014-1177-z 24771040

[37] Zięba G, Fox MG, Copp GH (2010) The effect of elevated temperature on spawning frequency and spawning behaviour of introduced pumpkinseed *Lepomis gibbosus* in Europe. J Fish Biol 77: 1850–1855. 10.1111/j.1095-8649.2010.02778.x 21078094

[38] Fobert E, Fox MG, Ridgway M, Copp GH. (2011) Heated competition: how climate change will affect competing non-native pumpkinseed and Eurasian perch in the U.K. J Fish Biol 79: 1592–1607. 10.1111/j.1095-8649.2011.03083.x 22136241

[39] Moran R, Harvey I, Moss B, Feuchtmayr H, Hatton K, Heyes T, et al (2010) Influence of simulated climate change and eutrophication on three-spined stickleback populations: a large scale mesocosm experiment. Freshw Biol 55: 315–325.

[40] Li Y, Liu X, Li X, Petitpierre B, Guisan A. (2014) Residence time, expansion toward the equator in the invaded range and native range size matter to climatic niche shifts in non-native species. Glob Ecol Biogeogr 23: 1094–1104.

[41] Williams P, Whitfield M, Biggs J, Bray S, Fox G, Nicolet P, et al (2003) Comparative biodiversity of rivers, streams, ditches and ponds in an agricultural landscape in Southern England. Conserv Biol 115: 329–341.

[42] Stakėnas S, Copp GH, Scott DM (2009) Tagging effects on three non-native fish species in England (*Lepomis gibbosus*, *Pseudorasbora parva*, *Sander lucioperca*) and of native *Salmo trutta* . Ecol Freshw Fish 18: 167–176.

[43] Peig J, Green AJ (2010) The paradigm of body condition: a critical reappraisal of current methods based on mass and length. Funct Ecol 24: 1323–1332.

[44] Copp GH, Stakėnas S, Cucherousset J (2010) Aliens vs. the natives: interactions between introduced *Lepomis gibbosus* and indigenous *Salmo trutta* in small streams of southern England In: Gido KB, Jackson D, editors. Community Ecology of Stream Fishes: Concepts, Approaches and Techniques. American Fisheries Society, Bethesda: Maryland pp. 347–370.

[45] Stakėnas S, Vilizzi L, Copp GH (2013) Habitat use, home range, movements and interactions of introduced *Lepomis gibbosus* and native *Salmo trutta* in a small stream of Southern England. Ecol Freshw Fish 22: 202–215.

[46] De Bono A, Peduzzi P, Kluser S, Giuliani G (2004) Impacts of summer 2003, United Nations Environment Programme.

[47] Rogers MW, Allen MS (2009) Exploring the generality of recruitment hypothesis for largemouth bass along a latitudinal gradient of Florida lakes. Trans Am Fish Soc 138: 23–37.

[48] Fox MG, Vila-Gispert A, Copp GH (2007) Life-history traits of introduced Iberian pumpkinseed *Lepomis gibbosus* relative to native populations. Can differences explain colonization success? J Fish Biol 71: 56–69.

[49] Danylchuk A, Fox MG (1994) Age and size dependent variation in the seasonal timing and probability of reproduction among mature female pumpkinseed. Environ Biol Fishes 39: 119–127.

[50] Miller SJ, Storck T (1982) Daily growth rings in otoliths of young-of-the-year largemouth bass. Trans Am Fish Soc 111: 527–530.

[51] Ludsin SA, DeVries DR (1997) First-year recruitment of largemouth bass: The interdependency of early life stages. Ecol Appl 7: 1024–1038.

[52] Garvey JE, Herra TP, Legget WC (2002) Protracted reproduction in sunfish: The temporal dimensions in fish recruitment revisited. Ecol Appl 12: 194–205.

[53] Copp GH (1990) Recognition of cohorts and growth of larval and juvenile roach *Rutilus rutilus* (L.), using size class ordination of developmental steps. J Fish Biol 36: 803–819.

[54] Divino JN, Tonn WM (2007) Effects of reproductive timing and hatch date on fathead minnow recruitment. Ecol Freshw Fish 16: 165–176.

[55] Bernard G, Fox M (1997) Effects of body size and population density on overwinter survival of age-0 pumpkinseeds. N Am J Fish Manage 17: 581–590.

[56] Murphy SC, Collins NC, Doka SE (2012) The effects of cool and variable temperatures on the hatch date, growth and overwinter mortality of a warmwater fish in small coastal embayments of Lake Ontario. J Great Lakes Res 38: 404–412.

[57] Copp GH, Fox MG, Kováč V (2002) Growth, morphology and life history traits of a cool-water European population of pumpkinseed *Lepomis gibbosus* . Arch Hydrobiol 155: 585–614.

[58] Persson L, Eklöv P (1995) Prey refuges affecting interactions between piscivorous perch and juvenile perch and roach. Ecology 76: 70–81.

[59] Persson L, Claessen D, De Roos AM, Byström P, Sjögren S, Svanbäch R, et al (2004) Cannibalism in a size-structured population: energy extraction and control. Ecol Monogr 74: 135–157.

[60] Fox MG, Keast A (1991) Effect of overwinter mortality on reproductive life history characteristics of pumpkinseed (*Lepomis gibbosus*) populations. Can J Fish Aquat Sci 48: 1792–1799.

[61] Keast A (1968) Feeding of some Great Lakes fishes at low temperatures. J Fish Res Board Can 25: 1199–1218.

[62] Post JR, Evans DO (1989) Size-dependent overwinter mortality of young-of-the-year yellow perch (*Perca flavescens*): laboratory, in situ enclosure, and field experiments. Can J Fish Aquat Sci 46: 1958–1968.

[63] Johnson TB, Evans DO (1996) Temperature constraints on overwinter survival of age-0 white perch. Trans Am Fish Soc 125: 466–471.

[64] Fox MG, Crivelli AJ (2001) Life history traits of pumpkinseed (*Lepomis gibbosus*) populations introduced into warm thermal environments. Arch Hydrobiol 150: 561–580.

[65] Dembski S, Masson G, Monnier D, Wagner P, Pihan JC (2006) Consequences of elevated temperatures on life-history traits of an introduced fish, pumpkinseed *Lepomis gibbosus* . J Fish Biol 69: 331–346.

