# Supplementary material for: How Will Climate Warming Affect Non-Native Pumpkinseed Lepomis gibbosus Populations in the U.K.?
Source: PLoS One. 2015 Aug 24;10(8):e0135482. doi: 10.1371/journal.pone.0135482 (PMC4547721; doi:10.1371/journal.pone.0135482)
Supplement: S1 File — Fig A in S1 File. Illustrates the total length-frequency distributions for young-of-the-year pumpkinseed reared in ambient and heated experimental ponds (England) at the end of phases II and III of the experiment. Table A in S1 File. Presents the number and total weight of YOY pumpkinseed recovered in an ambient and heated pond following the over-winter period, including mean, minimum and maximum total length and weight values. Table B in S1 File. Presents the results of Analysis of Covariance tests for differences in body condition between young-of-the-year pumpkinseed recovered from ambient temperature and heated ponds in March 2010. (DOCX) [file pone.0135482.s001.docx]

**Supporting Information – S1 file:** Over-winter study of recovered pumpkinseed

# Procedures

To examine over-winter survival of the pumpkinseed recovered from the main study, representative individuals of the YOY recovered at the end of Phase II were over-wintered. Over-wintering was restricted to two ponds, as four of the six ponds used in the main study were unavailable. These over-winter results do provide an indication of the effect that larger body size of the earlier-born individuals from the elevated temperature ponds may have on their survival and recruitment. However, because of the lack of replication, any differences between the two over-wintered populations should be interpreted with caution, as factors other than temperature could have affected the survival and recruitment of one or both populations.

As previously noted, Phase II of the main study was terminated on 14 October 2009, at which time the ponds were drained, and all recovered YOY were held separately in aerated containers (one per pond). To standardize the number of YOY reared over-winter and to minimize damage and stress, a random sub-sample (mean = 57  specimens per pond) was taken from each container, euthanized, and subsequently measured (*L*_T_, nearest mm) and weighed (nearest 0.01 g).  Sub-samples of the remaining specimens from the same treatment were pooled and stocked into a single ambient or elevated temperature pond for over-wintering. Based on the mean weights of the euthanized sub-sample, 61 g of ambient temperature YOY and 165 g of elevated temperature YOY were randomly selected from among the non-euthanized individuals, and stocked into their respective treatment ponds on 15 October 2009. The biomass used in each treatment provided an estimated 350 individuals for the over-winter experiment.

YOY were not provided with supplementary food during the over-winter period. No YOY were observed at the surface, indicating that hypoxia was not a factor in the study. The two ponds were drained on 16 March 2010, and YOY pumpkinseed were collected, euthanized, preserved and subsequently measured for *L*_T_ and weight. These data were used with the pre-winter measurement of YOY to identify changes in size-class distribution over the winter period.

Differences between thermal regimes in over-winter survivorship were reported as the percentage of individuals recovered in March 2010, relative to the 350 individuals that were estimated to have been stocked prior to winter. These differences could not be tested statistically because there were no replicate ponds for this part of the study; however, length-frequency plots of YOY stocked in the ponds before and after the winter period were used to help interpret over-winter survival differences between the two thermal regimes. We also used post-winter comparisons between treatments in body condition to infer over-winter changes that may have influenced survival. For this analysis, we used Analysis of Covariance on log_e_-transformed *L*_T_ and weight data on YOY recovered prior to winter (October 2009) and after winter (March 2010). In both cases, we used weight of the fish as the dependent variable, length as the covariate and the interaction between length and treatment as variables in the model.

**Results**

Approximately 93% of the YOY produced in a heated pond survived until the following spring (Table A). In contrast, only 34% of the YOY produced in an ambient temperature pond survived the over-winter period. Inspection of length-frequency plots of YOY from each treatment before and after the over-winter period (Fig. A) suggests that minimal somatic growth occurred during this period under either thermal regime, and that over-winter mortality was particularly severe on the smallest (8–15 mm *L*_T_) individuals in the ambient pond. A calculation based on the bimodal distribution of YOY before and after the over-winter period suggests that the over-winter survival rate for the small YOY in the ambient pond was ≈ 25%, whereas it increased to ≈ 36% for the larger YOY. In contrast, not only were there no individuals 8–15 mm *L*_T_ collected from the heated pond prior to or after the over-winter period, but also the mortality rate of the smallest YOY from that treatment (17–23 mm *L*_T_) was considerably lower than that of equivalent size over-wintered in the ambient pond.

**Table A.** Number (*n*) and total weight of YOY pumpkinseed recovered in an ambient and heated pond following the over-winter period (October 2009 to March 2010), along with their mean, minimum and maximum total length (*L*_T_) and weight. The number of individuals stocked for the over-winter experiment was standardized at 350, based on mean weights of a sub-set of individuals recovered at the end of Phase II of the study.

| Time period |  |  | Weight | *L*_T_ (mm) | | | Weight (g) | | |
| --- | --- | --- | --- | --- | --- | --- | --- | --- | --- |
| Temp. |  | *n* | (g) | Mean | Min. | Max. | Mean | Min. | Max. |
|  | | | | | | | | | |
| Ambient |  | 118 | 21.66 | **22^§^** | 14 | 35 | **0.18**^¶^ | 0.05 | 0.73 |
| Heated |  | 325 | 155.46 | **29^§^** | 17 | 47 | **0.48**^¶^ | 0.08 | 2.14 |
| Totals and means: | | 443 | 177.12 | 27 |  |  | 0.40 |  |  |
| Mean values in bold and annotated with the same symbol are significantly different (Students’ *t*-test, *P* < 0.0001). | | | | | | | | | |

**Fig. A. Total length-frequency distributions for young-of-the-year pumpkinseed reared in ambient and heated experimental ponds (England).** A) end of Phase II (October 2009); and B) end of Phase III (March 2010). Note the 10× difference in y-axis for post winter (B) fish number.

Individuals recovered from the ambient pond in March were significantly thinner than those collected from the heated pond (Table B). There was also a significant length × treatment interaction in these post-winter YOY, suggesting that the body condition difference between treatments was size-related. Inspection of length-weight plots showed that small YOY over-wintered in the ambient pond were in poorer condition in spring than those over-wintered in the heated pond, whereas the reverse was true in the larger-bodied YOY.


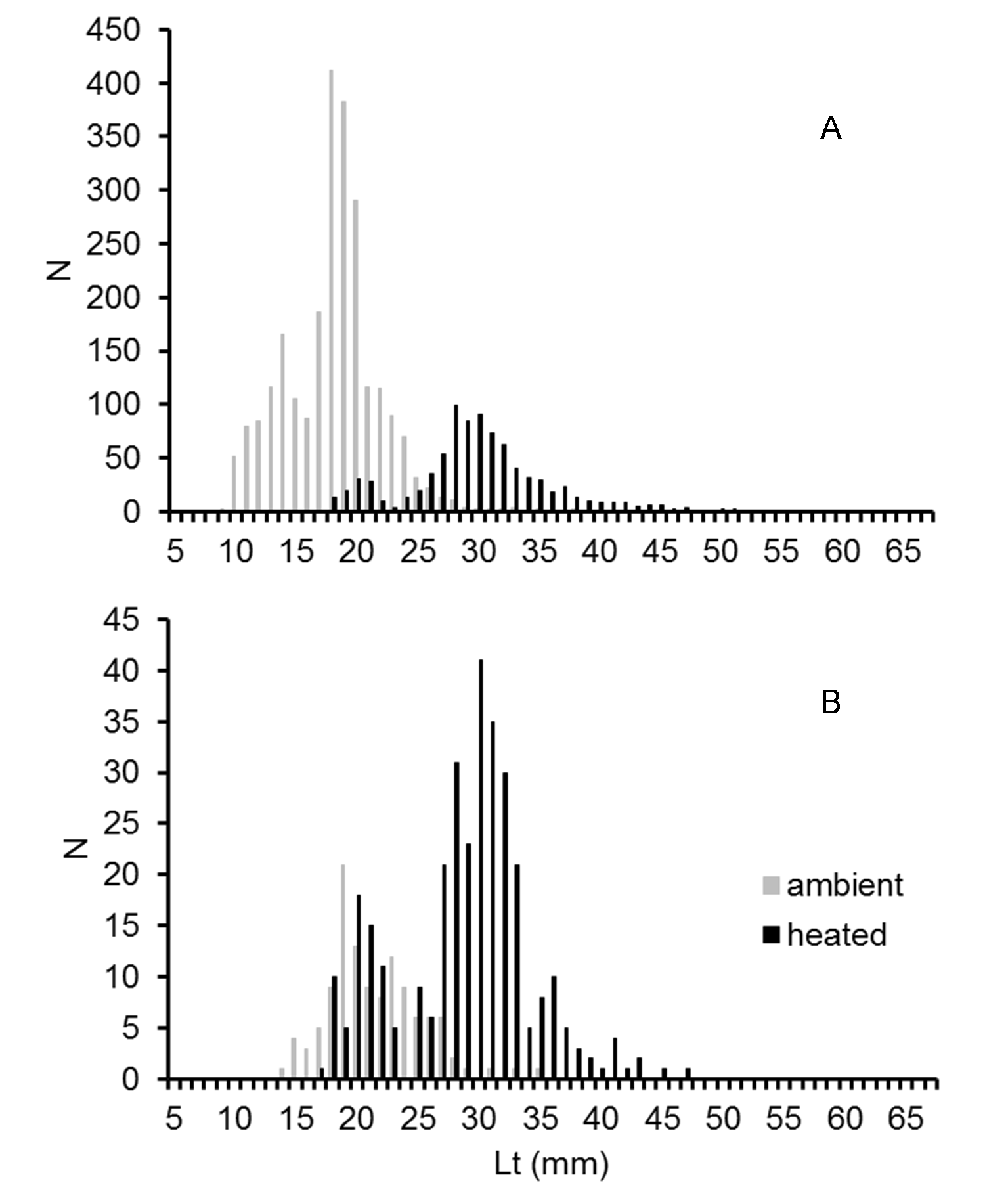


**Table B.** Results of Analysis of Covariance, testing for differences in body condition (wet weight relative to total length) between young-of-the-year pumpkinseed recovered from ambient temperature and heated ponds in March 2010. Total length and weight were log_e_-transformed prior to analysis.

| Effects | df | *F* | *P* |
| --- | --- | --- | --- |
| length | 1, 439 | 8302 | < 0.001 |
| temperature treatment | 1, 439 | 17.2 | < 0.001 |
| length × treatment | 1, 439 | 21.3 | < 0.001 |
